# 3-(2-Pyridylaminocarbonyl)propanoic acid

**DOI:** 10.1107/S1600536809016870

**Published:** 2009-05-14

**Authors:** Cheng-Feng Wang

**Affiliations:** a College of Chemistry & Bioengineering, Changsha University of Science & Technology, Changsha 410076, People’s Republic of China

## Abstract

In the crystal structure of the title compound, C_9_H_10_N_2_O_3_, the mol­ecules are linked by inter­molecular O—H⋯N and N—H⋯O hydrogen bonds, resulting in chains propagating in [010]. Weak intra­molecular and inter­molecular C—H⋯O inter­actions are also observed.

## Related literature

For background on the pharmaceutical applications of this family of compounds, see: Narendar *et al.* (2003 ▶); Ravlee *et al.* (2003 ▶).
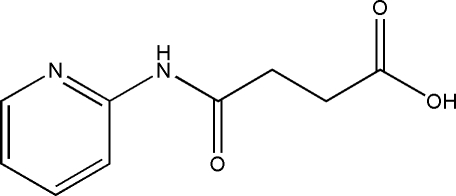


## Experimental

### 

#### Crystal data

C_9_H_10_N_2_O_3_
*M* *_r_* = 194.19
Monoclinic, 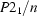

*a* = 12.7384 (10) Å
*b* = 5.0485 (5) Å
*c* = 13.8463 (12) Å
β = 92.924 (8)°
*V* = 889.29 (14) Å^3^
*Z* = 4
Mo *K*α radiation
μ = 0.11 mm^−1^
*T* = 113 K
0.22 × 0.04 × 0.03 mm

#### Data collection

Rigaku Saturn diffractometer
Absorption correction: multi-scan (*CrystalClear*; Rigaku, 2005 ▶) *T*_min_ = 0.995, *T*_max_ = 0.996
8079 measured reflections
1972 independent reflections
1297 reflections with *I* > 2σ(*I*)
*R*_int_ = 0.074

#### Refinement

*R*[*F* ^2^ > 2σ(*F* ^2^)] = 0.048
*wR*(*F* ^2^) = 0.104
*S* = 0.97
1972 reflections
135 parameters
1 restraint
H atoms treated by a mixture of independent and constrained refinement
Δρ_max_ = 0.23 e Å^−3^
Δρ_min_ = −0.22 e Å^−3^

### 

Data collection: *CrystalClear* (Rigaku, 2005 ▶); cell refinement: *CrystalClear*; data reduction: *CrystalClear*; program(s) used to solve structure: *SHELXS97* (Sheldrick, 2008 ▶); program(s) used to refine structure: *SHELXL97* (Sheldrick, 2008 ▶); molecular graphics: *SHELXTL* (Sheldrick, 2008 ▶); software used to prepare material for publication: *CrystalStructure* (Rigaku, 2005 ▶).

## Supplementary Material

Crystal structure: contains datablocks I, New_Global_Publ_Block. DOI: 10.1107/S1600536809016870/hb2960sup1.cif

Structure factors: contains datablocks I. DOI: 10.1107/S1600536809016870/hb2960Isup2.hkl

Additional supplementary materials:  crystallographic information; 3D view; checkCIF report

## Figures and Tables

**Table 1:** Hydrogen-bond geometry (Å, °)

*D*—H⋯*A*	*D*—H	H⋯*A*	*D*⋯*A*	*D*—H⋯*A*
O1—H1⋯N2^i^	0.954 (19)	1.744 (19)	2.690 (2)	170.4 (17)
N1—H1*A*⋯O2^ii^	0.96 (2)	1.86 (2)	2.824 (2)	176.6 (19)
C6—H6⋯O3	0.95	2.31	2.893 (2)	119
C3—H3*A*⋯O2^ii^	0.99	2.60	3.445 (2)	143
C3—H3*B*⋯O3^iii^	0.99	2.58	3.408 (2)	141
C7—H7⋯O3^iv^	0.95	2.56	3.319 (2)	137

Symmetry codes: (i) 
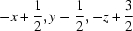
; (ii) 
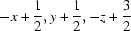
; (iii) 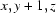; (iv) 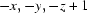.

**Fig. 1.**
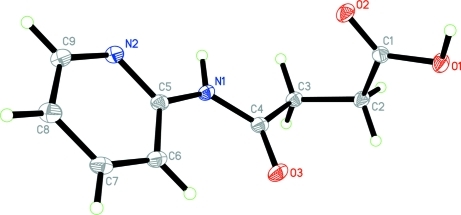
A view of the molecular structure of (I). Displacement ellipsoids are drawn at the 30% probability level (arbitrary spheres for H atoms).

**Fig. 2.**
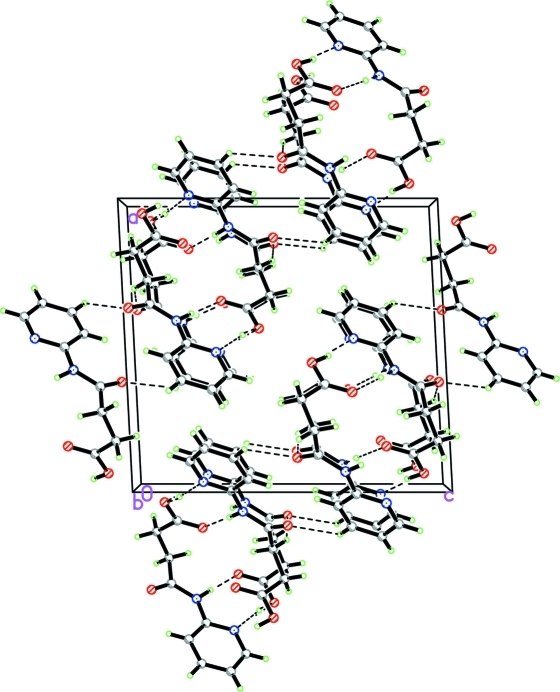
Part of the crystal structure of (I), with hydrogen bonds shown as dashed lines.

## supplementary crystallographic information

### Comment

Pyridine derivatives substituted by *N*-alkylation show useful pharmaceutical properties (Narendar *et al.*, 2003; Ravlee *et al.*, 2003). In this paper, the structure of 4-oxo-4-(pyridin-2-ylamino)butanoic acid (I), is reported which was synthesized by acylating reation of pyridin-2-amine with pyrrolidine-2,5-dione. The pyridin ring system is essentially planar with mean deviations of 0.0013 Å. In addition, there are C—H···O interactions, as shown in Fig. 2 and detailed in Table 1.

### Experimental

A solution of pyrrolidine-2,5-dione (1.0 g,10 mmol) in dimethylformamide (15 ml) was stirred at room temperature for 10 min. Pyridin-2-amine (0.94 g, 10 mmol) was added and the mixture was stirred for a further 3 h at 353 K. The resulting mixture was then poured into water (100 ml), yielding a white precipitate. The solid product was filtered off, washed with cold water and recrystallized from methnol, giving crystals of the title compound [yield: 1.17 g (61.4%)]. These were dissolved in mixture of methnol (10 ml) and water (3 ml) and the solution was kept at room temperature for 18 d. Natural evaporation of the solution gave colourless prisms of (I) (m.p. 454–455 K).

### Refinement

The O- and N-bound H atoms were located in a difference map and their positions and U_iso_ values were freely refined. The C-bound H atoms were geometrically placed (C—H = 0.95–0.99Å) and refined as riding with *U*_iso_(H) = 1.2*U*_eq_(C).

### Crystal data

**Table:**

C_9_H_10_N_2_O_3_	*F*(000) = 408
*M**_r_* = 194.19	*D*_x_ = 1.450 Mg m^−^^3^
Monoclinic, *P*2_1_/*n*	Melting point = 454–455 K
Hall symbol: -P 2yn	Mo *K*α radiation, λ = 0.71070 Å
*a* = 12.7384 (10) Å	Cell parameters from 2970 reflections
*b* = 5.0485 (5) Å	θ = 2.1–27.2°
*c* = 13.8463 (12) Å	µ = 0.11 mm^−^^1^
β = 92.924 (8)°	*T* = 113 K
*V* = 889.29 (14) Å^3^	Prism, colourless
*Z* = 4	0.22 × 0.04 × 0.03 mm

### Data collection

**Table:**

Rigaku Saturn diffractometer	1972 independent reflections
Radiation source: rotating anode	1297 reflections with *I* > 2σ(*I*)
confocal	*R*_int_ = 0.074
Detector resolution: 14.63 pixels mm^-1^	θ_max_ = 27.2°, θ_min_ = 2.1°
ω and φ scans	*h* = −16→16
Absorption correction: multi-scan (*CrystalClear*; Rigaku, 2005)	*k* = −6→6
*T*_min_ = 0.995, *T*_max_ = 0.996	*l* = −17→17
8079 measured reflections	

### Refinement

**Table:**

Refinement on *F*^2^	Secondary atom site location: difference Fourier map
Least-squares matrix: full	Hydrogen site location: inferred from neighbouring sites
*R*[*F*^2^ > 2σ(*F*^2^)] = 0.048	H atoms treated by a mixture of independent and constrained refinement
*wR*(*F*^2^) = 0.104	*w* = 1/[σ^2^(*F*_o_^2^) + (0.0361*P*)^2^] where *P* = (*F*_o_^2^ + 2*F*_c_^2^)/3
*S* = 0.97	(Δ/σ)_max_ = 0.001
1972 reflections	Δρ_max_ = 0.23 e Å^−^^3^
135 parameters	Δρ_min_ = −0.22 e Å^−^^3^
1 restraint	Extinction correction: *SHELXL*, Fc^*^=kFc[1+0.001xFc^2^λ^3^/sin(2θ)]^-1/4^
Primary atom site location: structure-invariant direct methods	Extinction coefficient: 0.041 (3)

### Special details

**Table:**

Geometry. All e.s.d.'s (except the e.s.d. in the dihedral angle between two l.s. planes) are estimated using the full covariance matrix. The cell e.s.d.'s are taken into account individually in the estimation of e.s.d.'s in distances, angles and torsion angles; correlations between e.s.d.'s in cell parameters are only used when they are defined by crystal symmetry. An approximate (isotropic) treatment of cell e.s.d.'s is used for estimating e.s.d.'s involving l.s. planes.
Refinement. Refinement of *F*^2^ against ALL reflections. The weighted *R*-factor *wR* and goodness of fit *S* are based on *F*^2^, conventional *R*-factors *R* are based on *F*, with *F* set to zero for negative *F*^2^. The threshold expression of *F*^2^ > σ(*F*^2^) is used only for calculating *R*-factors(gt) *etc*. and is not relevant to the choice of reflections for refinement. *R*-factors based on *F*^2^ are statistically about twice as large as those based on *F*, and *R*- factors based on ALL data will be even larger.

### Fractional atomic coordinates and isotropic or equivalent isotropic displacement parameters (Å^2^)

**Table:**

	*x*	*y*	*z*	*U*_iso_*/*U*_eq_	
O1	0.44178 (10)	0.2594 (3)	0.58044 (9)	0.0272 (4)	
H1	0.4642 (15)	0.145 (4)	0.6323 (13)	0.041*	
O2	0.34511 (10)	0.4177 (3)	0.69759 (9)	0.0290 (4)	
O3	0.12611 (10)	0.3483 (3)	0.52083 (9)	0.0284 (4)	
N1	0.08391 (12)	0.5393 (3)	0.66376 (11)	0.0216 (4)	
N2	−0.02347 (12)	0.4254 (3)	0.78432 (11)	0.0231 (4)	
C1	0.37059 (14)	0.4218 (4)	0.61433 (13)	0.0221 (4)	
C2	0.32399 (15)	0.6117 (4)	0.54040 (13)	0.0239 (5)	
H2A	0.3090	0.5171	0.4786	0.029*	
H2B	0.3753	0.7541	0.5288	0.029*	
C3	0.22283 (14)	0.7334 (4)	0.57436 (13)	0.0227 (5)	
H3A	0.2367	0.8194	0.6380	0.027*	
H3B	0.1972	0.8703	0.5277	0.027*	
C4	0.14010 (14)	0.5211 (4)	0.58271 (13)	0.0223 (5)	
C5	0.00578 (14)	0.3716 (4)	0.69450 (13)	0.0214 (5)	
C6	−0.04012 (15)	0.1661 (4)	0.63919 (13)	0.0244 (5)	
H6	−0.0190	0.1317	0.5756	0.029*	
C7	−0.11649 (15)	0.0153 (4)	0.67911 (14)	0.0265 (5)	
H7	−0.1484	−0.1261	0.6431	0.032*	
C8	−0.14707 (15)	0.0686 (4)	0.77148 (13)	0.0276 (5)	
H8	−0.1999	−0.0338	0.7999	0.033*	
C9	−0.09839 (15)	0.2750 (4)	0.82096 (13)	0.0256 (5)	
H9	−0.1190	0.3126	0.8845	0.031*	
H1A	0.1087 (16)	0.673 (4)	0.7090 (14)	0.043 (6)*	

### Atomic displacement parameters (Å^2^)

**Table:**

	*U*^11^	*U*^22^	*U*^33^	*U*^12^	*U*^13^	*U*^23^
O1	0.0313 (8)	0.0289 (8)	0.0218 (8)	0.0121 (7)	0.0044 (6)	0.0043 (6)
O2	0.0352 (8)	0.0350 (9)	0.0172 (7)	0.0102 (7)	0.0057 (6)	0.0054 (6)
O3	0.0363 (9)	0.0269 (8)	0.0221 (8)	0.0033 (6)	0.0026 (6)	−0.0067 (6)
N1	0.0237 (9)	0.0228 (10)	0.0185 (8)	−0.0006 (7)	0.0019 (7)	−0.0045 (7)
N2	0.0259 (9)	0.0232 (9)	0.0202 (8)	−0.0010 (8)	0.0006 (7)	−0.0007 (7)
C1	0.0223 (10)	0.0217 (11)	0.0222 (10)	−0.0005 (9)	0.0007 (8)	−0.0010 (9)
C2	0.0282 (11)	0.0246 (11)	0.0191 (10)	0.0051 (9)	0.0028 (8)	0.0020 (8)
C3	0.0274 (11)	0.0213 (11)	0.0191 (10)	0.0043 (9)	0.0000 (8)	0.0009 (8)
C4	0.0257 (11)	0.0208 (11)	0.0200 (10)	0.0074 (9)	−0.0031 (8)	0.0008 (8)
C5	0.0234 (11)	0.0214 (11)	0.0191 (10)	0.0037 (9)	−0.0021 (8)	0.0005 (8)
C6	0.0283 (12)	0.0237 (11)	0.0207 (10)	0.0042 (9)	−0.0033 (8)	−0.0034 (8)
C7	0.0299 (11)	0.0235 (12)	0.0252 (11)	0.0008 (9)	−0.0063 (9)	−0.0041 (9)
C8	0.0320 (12)	0.0238 (11)	0.0267 (11)	−0.0057 (9)	−0.0023 (9)	0.0030 (9)
C9	0.0311 (12)	0.0253 (12)	0.0203 (10)	−0.0022 (9)	0.0008 (8)	−0.0008 (9)

### Geometric parameters (Å, °)

**Table:**

O1—C1	1.326 (2)	C2—H2B	0.9900
O1—H1	0.954 (17)	C3—C4	1.512 (3)
O2—C1	1.214 (2)	C3—H3A	0.9900
O3—C4	1.229 (2)	C3—H3B	0.9900
N1—C4	1.365 (2)	C5—C6	1.400 (2)
N1—C5	1.390 (2)	C6—C7	1.373 (3)
N1—H1A	0.96 (2)	C6—H6	0.9500
N2—C9	1.340 (2)	C7—C8	1.382 (2)
N2—C5	1.344 (2)	C7—H7	0.9500
C1—C2	1.503 (2)	C8—C9	1.377 (2)
C2—C3	1.523 (2)	C8—H8	0.9500
C2—H2A	0.9900	C9—H9	0.9500
			
C1—O1—H1	107.1 (12)	H3A—C3—H3B	108.2
C4—N1—C5	128.65 (17)	O3—C4—N1	123.90 (19)
C4—N1—H1A	114.5 (12)	O3—C4—C3	121.78 (18)
C5—N1—H1A	116.3 (12)	N1—C4—C3	114.33 (16)
C9—N2—C5	118.17 (16)	N2—C5—N1	113.37 (16)
O2—C1—O1	123.11 (17)	N2—C5—C6	121.90 (18)
O2—C1—C2	122.87 (17)	N1—C5—C6	124.73 (17)
O1—C1—C2	114.02 (16)	C7—C6—C5	118.38 (18)
C1—C2—C3	110.98 (16)	C7—C6—H6	120.8
C1—C2—H2A	109.4	C5—C6—H6	120.8
C3—C2—H2A	109.4	C6—C7—C8	120.21 (18)
C1—C2—H2B	109.4	C6—C7—H7	119.9
C3—C2—H2B	109.4	C8—C7—H7	119.9
H2A—C2—H2B	108.0	C9—C8—C7	117.82 (18)
C4—C3—C2	109.99 (15)	C9—C8—H8	121.1
C4—C3—H3A	109.7	C7—C8—H8	121.1
C2—C3—H3A	109.7	N2—C9—C8	123.51 (18)
C4—C3—H3B	109.7	N2—C9—H9	118.2
C2—C3—H3B	109.7	C8—C9—H9	118.2
			
O2—C1—C2—C3	−16.5 (3)	C4—N1—C5—N2	171.25 (17)
O1—C1—C2—C3	163.40 (16)	C4—N1—C5—C6	−9.1 (3)
C1—C2—C3—C4	−64.77 (19)	N2—C5—C6—C7	−0.6 (3)
C5—N1—C4—O3	2.5 (3)	N1—C5—C6—C7	179.81 (16)
C5—N1—C4—C3	−177.43 (15)	C5—C6—C7—C8	0.5 (3)
C2—C3—C4—O3	−42.8 (2)	C6—C7—C8—C9	−0.2 (3)
C2—C3—C4—N1	137.14 (15)	C5—N2—C9—C8	−0.1 (3)
C9—N2—C5—N1	−179.95 (15)	C7—C8—C9—N2	0.0 (3)
C9—N2—C5—C6	0.4 (3)		

### Hydrogen-bond geometry (Å, °)

**Table:**

*D*—H···*A*	*D*—H	H···*A*	*D*···*A*	*D*—H···*A*
O1—H1···N2^i^	0.95 (2)	1.74 (2)	2.690 (2)	170 (2)
N1—H1A···O2^ii^	0.96 (2)	1.86 (2)	2.824 (2)	176.6 (19)
C6—H6···O3	0.95	2.31	2.893 (2)	119
C3—H3A···O2^ii^	0.99	2.60	3.445 (2)	143
C3—H3B···O3^iii^	0.99	2.58	3.408 (2)	141
C7—H7···O3^iv^	0.95	2.56	3.319 (2)	137

Symmetry codes: (i) −*x*+1/2, *y*−1/2, −*z*+3/2; (ii) −*x*+1/2, *y*+1/2, −*z*+3/2; (iii) *x*, *y*+1, *z*; (iv) −*x*, −*y*, −*z*+1.

## References

[bb1] Narendar, P., Parthiban, J. & Anbalagan, N. (2003). *Biol. Pharm. Bull* **26**, 182–187. 10.1248/bpb.26.182 12576677

[bb2] Ravlee, I., Sivakumar, R. & Muruganantham, N. (2003). *Chem. Pharm. Bull.* **51**, 162–170. 10.1248/cpb.51.162 12576649

[bb3] Rigaku (2005). *CrystalClear* and *CrystalStructure* Rigaku Corporation, Tokyo, Japan.

[bb4] Sheldrick, G. M. (2008). *Acta Cryst.* A**64**, 112–122. 10.1107/S0108767307043930 18156677

